# Assessment of Dengue and Chikungunya Infections among Febrile Patients Visiting Four Healthcare Centres in Yaoundé and Dizangué, Cameroon

**DOI:** 10.3390/v14102127

**Published:** 2022-09-27

**Authors:** Stella Mariette Nana-Ndjangwo, Borel Djiappi-Tchamen, Ruth Mony, Maurice Demanou, Joyce Keumezeu-Tsafack, Roland Bamou, Parfait Awono-Ambene, Charles Félix Bilong Bilong, Christophe Antonio-Nkondjio

**Affiliations:** 1 Laboratory of Parasitology and Ecology, Department of Animal Physiology and Ecology, Faculty of Science, University of Yaoundé I, Yaoundé P.O. Box 337, Cameroon; 2 Institut de Recherche de Yaoundé (IRY), Organisation de Coordination pour la lutte Contre les Endémies en Afrique Centrale (OCEAC), Yaoundé P.O. Box 288, Cameroon; 3 Vector Borne Diseases Laboratory of the Applied Biology and Ecology Research Unit (VBID-URBEA), Department of Animal Biology, Faculty of Science, University of Dschang, Dschang P.O. Box 067, Cameroon; 4 World Health Organization, IST West Africa, Ouagadougou P.O. Box 7019, Burkina Faso; 5 Department of Biological Sciences, University of Douala, Douala P.O. Box 24157, Cameroon; 6 Vector Biology Liverpool School of Tropical Medicine Pembroke Place, Liverpool L3 5QA, UK

**Keywords:** dengue, chikungunya, IgM, rtRT-PCR, Dizangué, Yaoundé, Cameroon

## Abstract

Dengue and chikungunya are now widely distributed in Cameroon, but there is still not enough information on their prevalence in different epidemiological settings. This study was undertaken to assess the prevalence of dengue and chikungunya in both urban and rural settings in Cameroon using three diagnostic tools. From December 2019 to September 2021, willing febrile (temperature >38 °C) outpatients visiting four healthcare facilities in the cities of Yaoundé and Dizangué were screened for dengue, and chikungunya. Clinical features of patient were recorded in a form, and their blood samples were analysed using real-time reverse transcriptase-polymerase chain reaction (rtRT-PCR), rapid diagnostic tests (RDTs) and enzyme-linked immuno-sorbent assays (ELISA). Odds ratios were used to determine the level of association between socio-demographic factors, clinical features, and infection status. The Kappa coefficient permitted to assess the level of agreement between RDTs and ELISA. Overall, 301 febrile patients were recruited in the study: 198 in Yaoundé and 103 in Dizangué. The prevalence of infection varied with the diagnostic tool used. For dengue diagnostics, 110 patients were positive to rtRT-PCR: 90 (45.45%) in Yaoundé, and 20 (19.42%) in Dizangué. The prevalence of dengue IgM using ELISA varied from 22.3% in Dizangué to 30.8% in Yaoundé. Dengue IgM rate using RDTs was 7.6% in Yaoundé and 3.9% in Dizangué. For chikungunya, one (0.5%) patient (Yaoundé, suburb) was positive to rtRT-PCR. The prevalence of chikungunya IgM according to ELISA varied from 18.4% in Dizangué to 21.7% in Yaoundé, while it was 4.5% in Yaoundé and 12.6% in Dizangué with RDTs. Only abdominal and retro-orbital pains were significantly associated with acute dengue infection. All four dengue serotypes were recorded, with a predominance of DENV-3 (35.45%) and DENV-4 (25.45%). Rapid Diagnostic Tests for either chikungunya or dengue displayed very poor sensitivity. This study further confirms the high endemicity of both dengue and chikungunya in Yaoundé and Dizangué. These data stress the need for active surveillance and the implementation of vector control measures to prevent the occurrence of outbreaks across the country.

## 1. Introduction

Dengue and chikungunya are major public health threats and responsible for high morbidity and/or mortality [1,2]. These viral diseases are on the rise and spreading to new areas. The number of dengue cases has increased by 30 times over the last 50 years [3,4], with more than 100 countries around the world presently endemic for dengue and/or chikungunya [1,2]. Both diseases are transmitted by *Aedes* species, particularly *Aedes aegypti* and *Aedes albopictus*, which are widely distributed across the world [5]. In Cameroon, dengue and chikungunya have been detected in many places across the country [6,7,8,9]. Although no major outbreaks of dengue or chikungunya have been reported in the country, frequent detection of dengue and chikungunya cases is common [10,11,12,13]. Dengue and chikungunya are caused by viruses (DENV and CHIKV) belonging to different families (*Flaviviridae* and *Togaviridae*, respectively) [2,14]. However, they display similar clinical symptoms, including fever, myalgia, headache, nausea, arthralgia, exanthema, and vomiting, making them difficult to distinguish in co-endemic areas [15,16]. Moreover from the fact that some of these clinical symptoms are similar to those of malaria, they are often largely overlooked, due to the hyperendemicity of the latter in Cameroon. Four distinct serotypes (DENV-1-4) are responsible for dengue infections, with a wide spectrum of clinical manifestations, including mild to severe signs that can evolve into a fatal outcome [17,18]. Chikungunya infections are rarely fatal but can lead to chronic illness and pains that can last for months or even years after the initial infection [19,20]. Four different CHIKV lineages have so far been identified: West African, East/Central/South African (ECSA), Asian Urban lineage (AUL), and Indian Ocean lineage (IOL), which emerged from an ECSA strain [21,22,23]. DENV and CHIKV diagnosis relies on methods directly targeting the virus or on serological assays detecting specific antibodies, namely Immunoglobulin M or G [4,24,25]. Real time reverse transcriptase-polymerase chain reaction (rtRT-PCR) is an excellent tool for early detection of dengue and chikungunya infections. This technique provides good results 3 to 7 days after onset of symptoms [4,26,27]. Serological assays, such as an enzyme linked immunosorbent assay (ELISA), cannot detect infection in the early stage, but provide good results from 5 days after the onset of symptoms (mostly fever). In addition to these tools, detection can be achieved using rapid diagnostics tests (RDTs) [25,28]. Despite being specific, the sensitivity of most of these tests was found to vary across studies and ecological settings [16,20]. 

In Cameroon, during recent years, even though the number of studies on either dengue or chikungunya has increased, the prevalence of these diseases is still not well documented. Recent studies indicate acute dengue–malaria co-infections in febrile patients and the presence of different dengue serotypes [13,29,30,31]. In urban and rural settings, serological studies established a close association between IgM and IgG (either for dengue or chikungunya) detection and some anthropological and environmental factors [6,32,33]. Limits in the routine diagnosis and management of both diseases by healthcare workers have also been reported in health care facilities in the city of Yaoundé [34].

The present study aimed to first assess the prevalence of dengue and chikungunya infections among outpatients visiting health care facilities in both urban and rural settings, and second to evaluate the performance of RDTs in the diagnosis of recent infections of both diseases. Such evaluations are critical for arboviral disease surveillance in Cameroon. 

## 2. Materials and Methods

### 2.1. Study Sites

This study was carried out in the city of Yaoundé (Centre region of Cameroon) in two hospitals of the urban zone (AD Lucem Obobogo and *Centre Médical d’Arrondissement* “CMA” of Mvog-Betsi), and in the Soa District Hospital at the city periphery of Yaoundé (urban zone). In the littoral region, the study was conducted in the Saint Joseph health care centre in the city of Dizangué (rural zone). Sampling sites characteristics are presented in Table 1. Samplings in Yaoundé were conducted from December 2019 to May 2021 and in Dizangué from June to September 2021. 

### 2.2. Study Design and Participants

**Figure 1:**
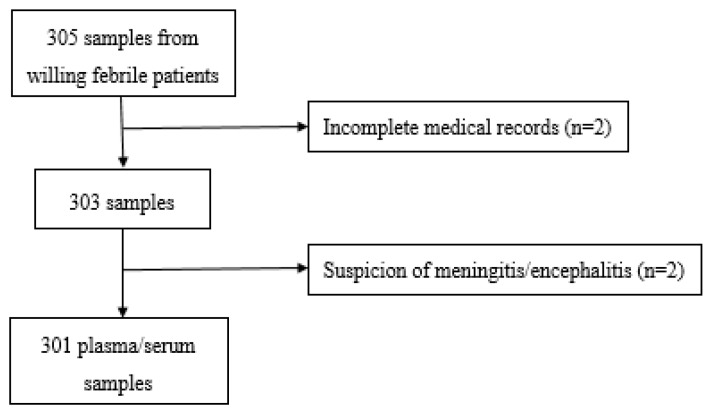
Flowchart of the study patient selection.

Patients of more than one year old with acute fever (temperature ≥38 °C) lasting for less than 7 days were eligible. Following a convenience sampling and after reading the study information note, outpatients meeting the eligibility criteria were included in the study by signing an informed consent form. However, patients with an incomplete medical record, as well as those with history of an acute injury or trauma and suspicion of meningitis/encephalitis, were excluded. The patient selection process is summarized in Figure 1.

### 2.3. Data Collection

#### 2.3.1. Sample Processing

Before the beginning of the study, one physician and one laboratory technician were recruited in each health care facility. They the study objectives and protocol were explained, and inclusion criteria and blood collection materials were provided to each health care facility. For each participant, a case report form (CRF) was used by health care workers to record both demographic (age, sex, residence, profession) and clinical information (fever onset date, signs and symptoms, yellow fever vaccination, travel history, malaria infection status, other particular symptoms). Blood sampling (from the median elbow vein) was done by an experienced lab technician using Vacutainer EDTA K_3_ tubes (Thermo Fisher Scientific, Illkirch-Graffenstaden, France). Plasma obtained after centrifugation or decantation was immediately used for RDT testing (dengue, chikungunya, malaria), then stored at −20 °C. Samples were collected weekly from the hospitals by an investigator and transported, while maintaining a cold chain, to the laboratory of the Institut de Recherche de Yaoundé (IRY) based at Organisation de Coordination pour la lutte contre les Endémies en Afrique Centrale (OCEAC), then stored at −20 °C for further analysis.

#### 2.3.2. Laboratory Analyses

In each health care facility laboratory, malaria diagnosis was performed using either HRP2/pLDH RDTs SD Bioline Malaria Ag Pf/Pan or Giemsa-stained thick blood smear. RDTs were performed for dengue diagnosis using SD Bioline Dengue Duo combo tests (Standard Diagnostic Inc., Gyeongii, Korea) and chikungunya diagnosis (Diagnostic Automation/Cortez Diagnostics, Woodland Hills, CA, USA). The SD Bioline dengue RDT simultaneously detects NS1 antigen, IgM, and IgG antibodies, while the chikungunya detects IgM and IgG antibodies.

At IRY’s laboratory, dengue and chikungunya IgM antibody detection was performed using EuroImmun Enzyme-Linked Immunosorbent Assay (ELISA) kits (EuroImmun, Lübeck, Germany). Analysis was performed following manufacturers’ instructions; samples displaying an optical density ≥1.1 were considered positive.

For molecular analysis, viral RNA extraction from plasma samples was conducted using a QIAmp Viral RNA Mini kit (Qiagen, Hilden, Germany), following the manufacturer’s instructions. Afterwards, real-time reverse transcriptase polymerase chain reaction (rt RT-PCR), according to protocols of the Centers for Disease Control and Prevention (CDC, Arbovirus Reference Collection, Fort Collins, CO, USA), was run for chikungunya virus detection, using the 6856 primers/probe set detecting both Asian and ECSA genotypes [35]. Dengue detection and serotyping was performed using the CDC DENV-1-4 Real Time RT-PCR Assay [36] CHIKV primers and probes sequences, DENV primers and probes kits, as well as DENV and CHIKV positive controls, were provided by the Centers for Disease Control and Prevention.

### 2.4. Case Definition

Case definition was defined as follows:

Positive CHIKV-rtRT PCR defined an acute CHIKV infection.

Positive DENV-rtRT PCR and/or positive NS1-RDT defined an acute DENV infection.

Positive DENV-IgM-ELISA and/or positive DENV-IgM-RDT defined a recent DENV infection.

Positive CHIKV-IgM-ELISA and/or positive CHIKV-IgM-RDT defined a recent CHIKV infection.

### 2.5. Statistical Analysis

Data collected from the CRF and laboratory analyses were recorded in a Microsoft Excel database (Microsoft Corp, Redmond, WA, USA) and double checked. Statistical analyses were performed with MedCalc and R version 3.6.3, at a significance level of α < 0.05. The sensitivity, specificity, positive, and negative predictive values (PPV and NPV), as well as their ninety-five percent confidence intervals, were generated for RDTs using the online MedCalc software, and using an ELISA assay as reference test. Furthermore, the level of agreement corresponding to the percentage of positive and negative results, in both diagnostic assays and a Kappa statistic, which ranges from 0 (two assays are completely independent) to 1 (two assays have perfect agreement), was calculated according to [37]. Logistic regressions were computed to obtain odd ratios, in order to determine the association between socio-demographic variables and infection status.

## 3. Results

### 3.1. Socio-Demographic Characteristics of the Study Population

Overall, 301 febrile patients aged from 1 to 77 years were screened during the study (198 in Yaoundé and 103 in Dizangué). The M/F sex ratio was 0.8 and most of the participants were students (n = 125, 41.53%), see Table 2. Among all participants, five (1.66%) reported to having received a yellow fever (YF) vaccine and thirty four (11.29%) had been out of their residence area during the previous month. Site status (urban or rural) was the only socio-demographic characteristic found to be significantly associated with recent dengue infection (*p* < 0.05) (Table 2). There was no significant association between any socio-demographic characteristics (sex, age, profession, YF vaccine, travelling history, or site status) and recent chikungunya infection (Table 2).

### 3.2. Clinical Features Associated with Acute Dengue and Chikungunya Infections

Patients with acute dengue infections were more likely to have abdominal pains (OR = 1.82, 95% CI: [1.05–3.16], *p* = 0.032) or retro-orbital pains (OR = 7.14, 95% CI [1.55–51.63], *p* = 0.021). These symptoms were sometimes associated with vomiting, cough, and malaria (Table 3). In contrast, no association was found between dengue infection and typhoid fever or acute inflammation (C-reactive protein (CRP) > 6 mg/L) (Table 3). For chikungunya, only one patient (0.5%) was detected as positive after RT-PCR, and this patient did not display any specific clinical symptoms except fever.

### 3.3. Monthly Distribution of Dengue Cases 

**Figure 2:**
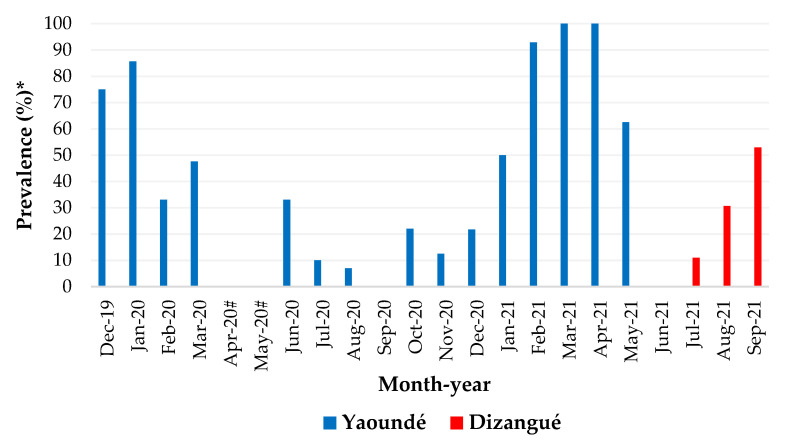
Prevalence of acute dengue infections in Yaoundé and Dizangué during the study period. * Cases confirmed by rtRT PCR. # No sample analysed due to COVID-19 lockdown.

In Yaoundé, the monthly distribution of dengue cases detected using rtRT-PCR indicated that the months of December 2019, January 2020, and January to May 2021 were those with the highest percentage (≥50% of analysed samples) of cases (Figure 2). During the months of April 2020 and May 2020, there were no samples collected, due to the Covid-19 lockdown. In Dizangué, the dengue prevalence varied from 0% (June 2021) to 52.9% (September 2021), with 11% and 30.7% during the months of July and August 2021, respectively (Figure 2). The only acute case of chikungunya detected by rtRT-PCR was recorded during the month of September 2020, in a Yaoundé suburb.

### 3.4. DENV Serotypes and Co-Infections

During the current study, dengue virus was detected in 110 patients: 90 (45.4%) in the city of Yaoundé and 20 (19.4%) in Dizangué (Table 4 and Table S1). Furthermore, all four dengue virus serotypes were detected. Among DENV rtRT-PCR positive samples, DENV-3 and DENV-4 serotypes were the most prevalent with 35.5% (n = 39) and 25.4% (n = 28) of cases, respectively. Some patients in Yaoundé (n = 23) were found to be infected with two serotypes, and one (n = 1) with three serotypes (Table 4). 

### 3.5. RDTs, ELISA, and rtRT-PCR Testing Results

All 301 plasma samples were tested with both RDTs and ELISA. For dengue, no sample was positive for NS1. Meanwhile, 53 samples were positive for DENV IgG using SD Bioline Duo RDTs (with 15.6%, n = 31 in Yaoundé and 21.3%, n = 22 in Dizangué). For chikungunya, 86 samples were positives for IgG using Cortez Diagnostics RDTs, with 33.8%, n = 67 in Yaoundé and 18.4%, n = 19 in Dizangué. There was no significant difference of DENV IgG prevalence recorded between Yaoundé and Dizangué (*p* > 0.05). On the contrary, the CHIKV IgG prevalence was significantly different according to these sites (*p* = 0.005) (Table 5).

The number of samples detected as positive (acute or recent infection) varied significantly according to the diagnostic tool. Only 19 patients were detected with DENV-IgM using SD Bioline RDTs (7.6%, n = 15 in Yaoundé and 3.9%, n = 4 in Dizangué). For chikungunya diagnostics, the number of patients positive to CHIKV-IgM was 22 when using Cortez Diagnostics RDTs (with 4.5%, n = 9 in Yaoundé and 12.6%, n = 13 in Dizangué).

The number of patients with DENV-IgM increased to 84 when ELISA was used (30.8%, n = 61 in Yaoundé and 22.3%, n = 23 in Dizangué). On the other hand, sixty two samples were IgM positive with EuroImmun ELISA kits (21.7%, n = 43 in Yaoundé and 18.4%, n = 19 in Dizangué).

With rtRT-PCR, 110 patients were detected as positive for DENV (45.4%, n = 90 in Yaoundé and 19.4%, n = 20 in Dizangué). One patient (0.5%) from Yaoundé suburb was detected as positive for CHIKV by rtRT-PCR (Table 5).

A comparison of the samples detected as positive or negative for IgM using RDTs and ELISA, irrespective of the study site, was conducted. For DENV, of the 84 IgM positive samples after ELISA, eight were positives using RDTs and 54 were negative (Table 6). Out of the 217 IgM negative samples after ELISA, 11 were positive for RDTs and 206 were negatives (Table 6). For CHIKV, of the 62 IgM positive samples to ELISA, eight were positives using RDTs. Meanwhile, of the 239 IgM negatives samples with ELISA, 14 were positive using RDTs and 225 were negative (Table 6).

### 3.6. Sensitivity and Specificity of RDTs Compared to ELISA

A EuroImmun ELISA kit for IgM detection assay was used as a reference, to assess the sensitivity and specificity of RDTs (SD Bioline and Cortez for IgM antibodies) in early detection of dengue and chikungunya. For dengue, the agreement was 71%, with a kappa statistic of 0.09. RDTs displayed a very low sensitivity 9.52% (95% CI: 4.20–17.91) and a specificity of 94.93% (95% CI: 91.11–97.44) (Table 7). 

For chikungunya, the agreement was 77%, with a kappa coefficient of 0.11. The sensitivity was 12.90% (95% CI: 5.74–23.85), with a specificity of 94.14% (95%CI: 90.37–96.76) (Table 7).

## 4. Discussion

The main objective of the current study was to assess dengue and chikungunya infections among febrile outpatients visiting healthcare centres in Yaoundé and Dizangué (Cameroon) using three different diagnostic tools: RDTs, ELISA, and RT-PCR. A high number of dengue infections were detected. The recorded prevalence of IgM and IgG antibodies was comparable to previous studies. In fact, some studies previously conducted in Cameroon indicated a prevalence of DENV IgM ranging from 0.1% to 0.3% and DENV IgG ranging from 9.8% to 61.4% [32]. For CHIKV, the prevalence of IgM in a rural site was 51.4% and 39% for IgG [6]. 

Dengue and chikungunya infections were more frequent in Yaoundé city centre and its periphery compared to the rural site of Dizangué. This could be due to the frequent samplings realized in Yaoundé compared to Dizangué, and probably to the presence of abundant arboviral vectors in urban sites [38,39]. Indeed, studies conducted previously reported a large distribution of both diseases’ main vectors *Aedes aegypti* and *Aedes albopictus* in urban and peri-urban areas [40,41,42]. Cameroon is also home to several arboviral and parasitic diseases [43], and due to the high number of symptoms shared by these diseases, reliable laboratory diagnosis are of paramount importance for disease surveillance and good clinical management [16,44,45].

Acute dengue infection was found to be significantly associated with abdominal and retro-orbital pains. Arboviral diseases such as dengue and chikungunya are commonly associated with an array of ocular manifestations, which can evolve into persistent visual impairment [46,47]. Others symptoms such as headache, myalgia, asthenia, or vomiting, which are common to other endemic diseases such as malaria or typhoid, were also recorded but were not significantly associated with dengue or chikungunya infections. The poor correlation between some symptoms and dengue or chikungunya infections could probably result from the co-endemicity of diseases displaying similar symptoms [20]. About 32% of dengue positive patients also had malaria, confirming the increasing co-endemicity of these two vector-borne diseases in Cameroon [13,29,30].

In Yaoundé, dengue cases were more frequent during the months of December to January 2020 and from January to May 2021. These periods coincide with the long dry season and part of the short rainy season. The frequent occurrence of dengue cases during these periods could be linked to the presence of suitable breeding opportunities for *Aedes* vectors during both the rainy and dry seasons [40]. In Dizangué, samplings were only conducted during four months (June to September 2021) and this sampling design did not permit assessing the seasonal variation of DENV and/or CHIKV transmission. Warmer temperatures were reported to be positively associated with dengue incidence in Mexico and China, by influencing the vector biology. Indeed, high temperatures increased mosquitoes’ feeding behaviour by hastening the blood digestion process, resulting in an increased likelihood of dengue transmission [48,49].

This study confirmed the simultaneous circulation of the four known dengue virus serotypes in the study sites, with a predominance of the DENV-3 and DENV-4 serotypes. Till recently, only three DENV serotypes (DENV-1, DENV-2 and DENV-3) were found in Cameroon, as well as in neighbouring countries (Gabon and Democratic Republic of Congo) [11,13,31,50,51]. DENV-4 serotype was detected in high frequency in patients with acute dengue infections. This first detection of DENV-4 serotype may have resulted from a recent introduction in the country following the intensification of trading exchanges between Cameroon and Asia. This serotype originating from Asia, where it is widespread, was also reported in South America and in very low frequency in some African countries [52].

Multitype serotype infections (concurrent infections in an individual with different serotypes of dengue virus) were also registered. According to Rao and others, multitype serotype infection could lead to Dengue Haemorrhagic Fever (DHF), but this requires further investigation [53].

The anti-DENV-IgM and anti-CHIKV-IgM prevalence were relatively high in both Yaoundé and Dizangué and this was in agreement with the probable frequent transmission of the disease, as suggested earlier [6,32]. High anti-DENV-IgG was also recorded and was consistent with previous viral exposure to dengue. The high CHIKV IgG frequency in rural area could have resulted from one or several epidemics, rather than endemic transmission [6,8,33]. During primary dengue infections, patients present detectable IgM from day 5 post-onset of symptoms and IgG after 8 to 10 days, whereas in secondary dengue infection, DENV IgG develops rapidly, with high titers soon after the onset of fever, while IgM can be undetectable [54]. From this analysis, it is likely that most of the recorded cases were primary dengue infections.

The SD Bioline Dengue Duo NS1 RDT did not provided a positive result for acute dengue infection (NS1 positive). This could have been due to the fact that most of the patients were screened 1 to 3 days after fever onset. Indeed, the NS1 part of the SD Bioline Duo RDTs has been reported to display low sensitivity during the early stage of dengue infection [55,56]. Furthermore, the low sensitivity of SD Bioline and Cortez Diagnostics RDTS compared to EuroImmun ELISA could have resulted from the misinterpretation of weak signals. This particular problem was highlighted in previous studies [57]. The investigations made to date indicated that health care workers in the field tend to read the results of RDT tests before the time recommended by the manufacturer [57,58], reducing by so doing the performance of RDTs. This situation points to the need of providing specific training to health care workers, to improve standards and good practices in the storage, diagnosis, and interpretation of RDTs tests. RDTs, despite some limits, are useful tools for clinicians in resource limited countries, as they quickly confirm the diagnosis of diseases, therefore contributing to an optimal clinical management of cases and avoiding unnecessary consumption of antimalarial or antibiotics drugs. For DENV and CHIKV RDTs, a relatively high specificity (94.93% and 94.14%, respectively) and % of agreement (71% and 77%) was recorded, but this data need further consideration, since several authors reported high rates of cross reactivity between dengue and chikungunya infection using EuroImmun ELISA kits. In a study undertaken in Brazil, the cross reactivity frequency increased up to 31.6% for patients with acute dengue, and it was 46.7% for patients with dengue IgM antibodies, which was frequent in dengue-endemic settings [20].

The present study has some limitations. The dengue viral serotypes were not sequenced; sequencing would have provided more detailed information on the origin of the viral genotypes found in the study. To better appraise diagnostic test performance, RDTs and ELISA results need to be confirmed using a plaque reduction neutralization test (PRNT), considered as one of the reference methods for serological assays. A second sample could have been collected (5 days or more post onset of fever), which may have improved the sensitivity of ELISA and RDTs based on the timing of sample collection.

## 5. Conclusions

This study indicated different prevalence patterns of dengue and chikungunya in Yaoundé and Dizangué. All four known dengue serotypes were recorded. These data highlight the need for urgent action to combat the disease. In Cameroon, dengue and chikungunya are still overlooked, due to the hyperendemicity of malaria. In the light of these data, more emphasis has to be put on the frequent diagnosis of these diseases in health care facilities. Combined measures, including vector control, community-sensitization, and improved diagnostics, need to be promoted, to effectively fight against these diseases. 

## Figures and Tables

**Table 1:** Characteristics of the health care facilities where the survey was undertaken.

Characteristics	Healthcare Centres			
	AD Lucem Obobogo	CMA Mvog-Betsi	Soa District Hospital	St Joseph Health Centre Dizangué
Region	Centre	Centre	Centre	Littoral
Health District	Efoulan	Biyem-assi	Soa	Edea
GPS coordinates	3°52′ N; 3°42′ N 11°25′ E; 11°32′ E	3°52′ 30′ N; 3°47′ 30′ N 11°27′ E; 11°30′30′ E	4°8′ N; 3°52′ N 11°31′ E; 11°41′ E	4°10′ N; 3°10′ N 10°30′ E; 9°40′ E
Health facility status	Confessional	Public	Public	Confessional
Population covered	252,501	268,428	69,084	32,627
Number of medical staff	27	43	65	12
Average number of consultation per week	87	93	325	40
Environment	Urban	Urban	Urban	Rural
Climatic seasons	4 seasons: 2 rainy and 2 dry	4 seasons: 2 rainy and 2 dry	4 seasons: 2 rainy and 2 dry	2 seasons: 1 rainy and 1 dry

**Table 2:** Association between demographic characteristics of the patients and recent infection with dengue and chikungunya.

Characteristics	All Participants (N = 301)	Dengue			Chikungunya		
	n	Recent Infection (%)	OR (95% CI)	*p* Value *	Recent Infection (%)	OR (95% CI)	*p* Value *
Gender							
Female	166	49 (29.5)	1.23 (0.74–2.07)	0.45	39 (23.5)	1	_
Male	135	46 (34)	1	_	37 (27.4)	1.23 (0.70–2.14)	0.50
Age							
<5	65	17 (26.1)	1	_	13 (20)	1	_
5–17	99	35 (35.3)	0.65 (0.32–1.29)	0.21	31 (31.3)	0.55 (0.26–1.15)	0.11
18−44	119	34 (28.6)	0.88 (0.44–1.75)	0.72	26 (21.8)	0.89 (0.41–1.88)	0.77
≥45	18	9 (50)	0.36 (0.12–1.08)	0.05	7 (38.9)	0.39 (0.13–1.28)	0.10
YF vaccination	5	2 (40)	1.31 (0.20–6.90)	0.71	3 (60)	1	_
Not vaccinated YF	296	93 (31.4)	1	_	73 (24.7)	4.55 (0.51–55.46)	0.10
Travelled last month	34	7 (20.6)	0.53 (0.19–1.31)	0.17	7 (20.6)	0.74 (0.26–1.86)	0.67
Didn’t travelled	267	88 (33)	1	_	69 (25.8)	1	_
Profession							
Civil servant	15	6 (40)	1	_	3 (20)	1	_
Small business	55	20 (36.4)	0.84 (0.24–2.79)	0.76	15 (27.3)	0.69 (0.14–2.61)	0.57
Student	125	43 (34.4)	1.28 (0.40–3.85)	0.66	36 (28.8)	0.64 (0.13–2.20)	0.47
Retired	3	1 (33.3)	1.25 (0.08–4.54)	0.82	1 (33.3)	0.50 (0.03–19.46)	0.61
Jobless	103	35 (34)	1.30 (0.40–3.98)	0.64	21 (20.4)	1.01 (0.20–3.60)	0.97
Site status							
Yaoundé (urban)	198	71 (35.8)	1.83 (1.07–3.19)	**0.02**	25 (12.6)	0.92 (0.53–1.61)	0.78
Dizangué (rural)	103	24 (23.3)	1	**_**	27 (26.2)	1	_

Recent DENV infection is a positive DENV-IgM-ELISA and/or positive DENV-IgM- RDT. Recent CHIKV infection is a positive CHIKV-IgM-ELISA and/or positive CHIKV-IgM-RDT. * Significative when *p* < 0.05 (bolded).

**Table 3:** Regression analysis assessing the association between clinical symptoms and acute dengue infection.

Characteristics	Tested (N = 301)	Acute * DENV Infection (n = 110)	Negative (n = 191)	OR (95%CI)	*p* Value ^#^
Clinical signs	n **	n ** (%)	n ** (%)		
Headache	178	70 (39.3)	108 (60.7)	0.78 (0.45–1.32)	0.36
Nausea	42	13 (31)	29 (69)	0.95 (0.43–2.06)	0.91
Vomiting	75	19 (25.3)	56 (74.7)	0.54 (0.28–0.99)	0.05
Abdominal pain	96	40 (41.7)	56 (58.3)	1.82 (1.05–3.16)	**0.03**
Rash	4	0 (0)	4 (100)	_	0.98
Myalgia	14	3 (21.4)	11 (78.6)	0.34 (0.07–1.26)	0.14
Arthralgia	31	11 (35.5)	20 (64.5)	0.76 (0.30–1.82)	0.55
Retro-orbital pain	9	7 (77.8)	2 (22.2)	7.14 (1.55–51.63)	**0.02**
Diarrhoea	37	10 (27)	27 (73)	0.55 (0.22–1.26)	0.17
Asthenia	62	16 (26.7)	46 (73.3)	0.64 (0.33–1.22)	0.18
Cough	12	7 (58.3)	5 (41.7)	3.44 (0.97–13.87)	0.06
Laboratory results					
Malaria	153	49 (32)	104 (68)	1.55 (0.97–2.52)	0.06
Typhoid fever	13	7 (53.8)	6 (46.2)	0.88 (0.04–10.16)	0.92
CRP above 6 mg/L	5	4 (80)	1 (20)	8.94 (0.81–308.89)	0.12

* Acute DENV infection was determined by rtRT-PCR for dengue virus detection and serotyping assay. ** Number of patients presenting a particular symptom or disease. ^#^ Significative when *p* < 0.05 (bolded).

**Table 4:** Dengue serotypes and co-infections by different serotypes, according to study site.

Sites	Tested		Serotypes				DENV Co-Infection				
		Positives (%)	DENV-1 (%)	DENV-2 (%)	DENV-3 (%)	DENV-4 (%)	1 + 2 (%)	2 + 3 (%)	2 + 4 (%)	3 + 4 (%)	2 + 3 + 4 (%)
Yaoundé	198	90	7	5	36	18	1	2	1	19	1
		(45.4)	(3.5)	(2.5)	(18.2)	(9.1)	(0.5)	(1)	(0.5)	(9.6)	(0.5)
Dizangué	103	20	3	4	3	10	_	_	_	_	_
		(19.4)	(2.9)	(3.9)	(2.9)	(9.7)					
Total	301	110	10	9	39	28	1	2	1	19	1
		(36.5)	(9.1)	(8.2)	(35.5)	(25.4)	(0.9)	(1.8)	(0.9)	(17.3)	(0.9)

**Table 5:** Diagnostic tool results, according to study site.

			Sites	
Diagnostic Tools	Virus	Target	Yaoundé n Positives (%)	Dizangué n Positives (%)
RDTs	DENV	NS1	0 (0)	0 (0)
	DENV	IgG	31 (15.6)	22 (21.3)
	CHIKV	IgG	67 (33.8)	19 (18.4)
	DENV	IgM	15 (7.6)	4 (3.9)
	CHIKV	IgM	9 (4.5)	13 (12.6)
ELISA	DENV	IgM	61 (30.8)	23 (22.3)
	CHIKV	IgM	43 (21.7)	19 (18.4)
rtRT-PCR	DENV	RNA	90 (45.4)	20 (19.4)
	CHIKV	RNA	1 (0.5)	0 (0)

**Table 6:** Results of ELISA crossed with those of RDTs for dengue and chikungunya early marker (IgM) detection.

ELISA IgM	RDTs IgM
Dengue	
Positive: 84	Positive: 8
	Negative: 76
Negative: 217	Positive: 11
	Negative: 206
Chikungunya	
Positive: 62	Positive: 8
	Negative: 54
Negative: 239	Positive: 14
	Negative: 225

**Table 7:** Agreement, sensitivity, specificity, PPV, and NPV of RDTs versus ELISA used as reference.

Tests	% Agreement with ELISA IgM (Kappa)	Sensitivity (95% CI)	Specificity (95% CI)	PPV (95% CI)	NPV (95% CI)
Dengue					
RDTs SD Duo (IgM)	71 (0.09)	9.52 (4.20–17.91)	94.93 (91.11–97.44)	42.11 (23.26–63.57)	73.05 (71.53–74.52)
Chikungunya					
RDTs Cortez (IgM)	77 (0.11)	12.90 (5.74–23.85)	94.14 (90.37–96.76)	36.36 (20.07–56.54)	80.65 (79.02–82.17)

% agreement = Percentage of agreement equal to the percentage of test results with both positive and with both negative responses and its corresponding Kappa statistic (the higher the better; see texts in statistical analysis). PPV = positive predictive value; NPV = negative predictive value.

## Data Availability

All data generated or analysed during this study are included in the published article and its Supplementary Information files.

## Supplementary Materials

The following supporting information can be downloaded at: https://www.mdpi.com/article/10.3390/v14102127/s1, Table S1: Characteristics of DENV and CHIKV RT-PCR positive participants recorded in the study.

## Abbreviations

CMA: *Centre Médical d’Arrondissement*. CRF: case report form. ELISA: enzyme-linked immuno-sorbant assay. RDTs: rapid diagnostic tests. rtRT-PCR: real-time reverse transcriptase-polymerase chain reaction
