# Supplementary material for: Assessment of Dengue and Chikungunya Infections among Febrile Patients Visiting Four Healthcare Centres in Yaoundé and Dizangué, Cameroon
Source: Viruses. 2022 Sep 27;14(10):2127. doi: 10.3390/v14102127 (PMC9612120; doi:10.3390/v14102127)
Supplement: Supplementary file 1 [file viruses-14-02127-s001.zip › viruses-1878550-supplementary.pdf]

Patient	age	sex	Serotype	Ct value
1	16	M	3	22.61
2	11	F	3	26.55
3	19	F	3	28.69
4	39	F	3	27.25
5	58	F	3	27.09
6	25	M	4	27.46
7	22	F	3	27.77
8	12	M	3 + 4	24.71 / 11.75
9	15	F	3	15.65
10	12	F	3	27.78
11	14	F	3	22.34
12	11	F	3	27.85
13	35	M	3 + 4	27.61 / 27.14
14	19	F	3	19.98
15	10	M	3	27.46
16	23	M	3 + 4	21.81 / 29.82
17	17	M	3	17.72
18	41	F	3	15.48
19	4	M	3	18.26
20	4	F	3	20.53
21	7	F	3 + 4	26.11 / 18.07
22	30	F	2 + 4	22.11 / 12.57
23	3	M	3	27.70
24	34	F	4	16.45
25	25	F	3 + 4	15.78 / 16.69
26	28	F	3 + 4	16.82 / 13.36
27	25	F	2 + 3	37.15 / 18.78
28	23	F	2	37.15
29	2	M	1	15.49
30	18	F	4	22.90
31	3	F	4	27.42
32	7	M	4	34.99
33	10	M	1	15.00
34	25	F	2	35.92
35	39	F	1	19.16
36	17	M	1	9.09
37	36	F	2	37.12
38	23	F	1	15.15
39	32	F	1	31.45
40	58	M	2	32.84
41	9	F	1 + 2	22.12 / 31.75
42	7	F	1	16.94
43	25	F	2	37.42
44	19	M	4	20.90
45	9	M	4	28.11
46	1	M	4	19.57

47	43	M	4	31.97
48	24	M	4	35.99
49	22	F	4	25.31
50	3	F	4	17.93
51	4	M	4	33.84
52	7	M	4	11.72
53	4	F	3 + 4	27.98 / 27.11
54	16	M	3	29.32
55	18	F	3 + 4	17.69 / 16.63
56	10	F	2 + 3 + 4	37.46 / 22.59 / 19.69
57	21	F	3	28.99
58	16	M	3	27.83
59	8	F	3	27.99
60	13	M	4	19.81
61	17	F	3 + 4	26.85 / 26.91
62	2	M	3	15.92
63	39	M	3	15.88
64	8	M	3 + 4	31.14 / 15.66
65	6	F	3	21.74
66	15	F	2 + 3	36.34 / 18.91
67	25	M	4	30.54
68	4	F	3 + 4	14.83 / 31.19
69	14	F	3 + 4	15.16 / 26.80
70	22	F	3	17.18
71	2	M	3 + 4	21.39 / 16.25
72	5	M	3 + 4	27.55 / 26.85
73	17	M	3	31.83
74	12	M	3 + 4	31.37 / 26.99
75	37	M	3	27.86
76	10	M	3	30.39
77	17	F	3 + 4	19.04 / 26.88
78	32	F	3 + 4	18.76 / 15.56
79	24	F	3 + 4	16.29 / 34.99
80	23	F	3	15.50
81	9	M	3	26.05
82	6	F	3	31.94
83	22	M	3	27.23
84	10	F	3	26.62
85	22	F	4	31.95
86	21	M	3	27.66
87	4	M	3	23.84
88	12	M	4	26.72
89	3	F	3	22.56
90	5	F	3 + 4	26.90 / 23.66
91	23	M	1	37.75
92	21	F	2	31.49
93	4	F	1	32.73
94	60	F	1	30.38

95	33	F	2	30.61
96	7	M	2	31.61
97	1	M	2	10.91
98	3	F	4	31.44
99	31	F	4	22.56
100	29	F	4	25.87
101	19	F	4	32.84
102	4	M	4	19.60
103	5	M	4	34.31
104	26	F	4	29.72
105	6	F	4	25.93
106	2	F	4	11.50
107	13	F	4	33.37
108	1	M	3	24.14
109	4	M	3	27.85
110	43	M	3	26.96

Site	Site status	Sampling month
Yaounde	Urban	December 2019
Yaounde	Urban	December 2019
Yaounde	Urban	December 2019
Yaounde	Urban	January 2020
Yaounde	Urban	January 2020
Yaounde	Urban	January 2020
Yaounde	Urban	January 2020
Yaounde	Urban	January 2020
Yaounde	Urban	January 2020
Yaounde	Urban	January 2020
Yaounde	Urban	January 2020
Yaounde	Urban	January 2020
Yaounde	Urban	January 2020
Yaounde	Urban	January 2020
Yaounde	Urban	February 2020
Yaounde	Urban	February 2020
Yaounde	Urban	January 2020
Yaounde	Urban	February 2020
Yaounde	Urban	March 2020
Yaounde	Urban	March 2020
Yaounde	Urban	March 2020
Yaounde	Urban	February 2020
Yaounde	Urban	February 2020
Yaounde	Urban	March 2020
Yaounde	Urban	March 2020
Yaounde	Urban	March 2020
Yaounde	Urban	March 2020
Yaounde	Urban	March 2020
Yaounde	Peri-urban	June 2020
Yaounde	Peri-urban	June 2020
Yaounde	Urban	June 2020
Yaounde	Urban	June 2020
Yaounde	Peri-urban	June 2020
Yaounde	Peri-urban	July 2020
Yaounde	Peri-urban	August 2020
Yaounde	Peri-urban	October 2020
Yaounde	Peri-urban	October 2021
Yaounde	Peri-urban	November 2020
Yaounde	Urban	December 2020
Yaounde	Urban	December 2020
Yaounde	Peri-urban	December 2020
Yaounde	Urban	December 2020
Yaounde	Urban	December 2020
Yaounde	Peri-urban	January 2021
Yaounde	Urban	January 2021
Yaounde	Peri-urban	January 2021

Yaounde	Peri-urban	January 2021
Yaounde	Urban	January 2021
Yaounde	Urban	January 2021
Yaounde	Urban	January 2021
Yaounde	Urban	January 2021
Yaounde	Peri-urban	January 2021
Yaounde	Peri-urban	January 2021
Yaounde	Peri-urban	January 2021
Yaounde	Peri-urban	January 2021
Yaounde	Peri-urban	February 2021
Yaounde	Peri-urban	February 2021
Yaounde	Peri-urban	February 2021
Yaounde	Peri-urban	February 2021
Yaounde	Peri-urban	February 2021
Yaounde	Peri-urban	February 2021
Yaounde	Peri-urban	February 2021
Yaounde	Urban	January 2021
Yaounde	Urban	February 2021
Yaounde	Peri-urban	February 2021
Yaounde	Peri-urban	February 2021
Yaounde	Peri-urban	February 2021
Yaounde	Peri-urban	February 2021
Yaounde	Peri-urban	February 2021
Yaounde	Peri-urban	March 2021
Yaounde	Peri-urban	March 2021
Yaounde	Peri-urban	March 2021
Yaounde	Peri-urban	March 2021
Yaounde	Peri-urban	March 2021
Yaounde	Peri-urban	March 2021
Yaounde	Peri-urban	March 2021
Yaounde	Peri-urban	March 2021
Yaounde	Peri-urban	March 2021
Yaounde	Peri-urban	March 2021
Yaounde	Peri-urban	March 2021
Yaounde	Peri-urban	April 2021
Yaounde	Peri-urban	April 2021
Yaounde	Peri-urban	April 2021
Yaounde	Peri-urban	April 2021
Yaounde	Peri-urban	April 2021
Yaounde	Peri-urban	May 2021
Yaounde	Peri-urban	May 2021
Yaounde	Peri-urban	May 2021
Yaounde	Peri-urban	May 2021
Yaounde	Peri-urban	May 2021
Dizangue	Rural	July 2021
Dizangue	Rural	July 2021
Dizangue	Rural	July 2021
Dizangue	Rural	August 2021

Dizangue	Rural	August 2021
Dizangue	Rural	August 2021
Dizangue	Rural	August 2021
Dizangue	Rural	August 2021
Dizangue	Rural	August 2021
Dizangue	Rural	August 2021
Dizangue	Rural	August 2021
Dizangue	Rural	September 2021
Dizangue	Rural	September 2021
Dizangue	Rural	September 2021
Dizangue	Rural	September 2021
Dizangue	Rural	September 2021
Dizangue	Rural	September 2021
Dizangue	Rural	September 2021
Dizangue	Rural	September 2021
Dizangue	Rural	September 2021
